# Supplementary material for: Non-neutralizing antibodies elicited by recombinant Lassa–Rabies vaccine are critical for protection against Lassa fever
Source: Nat Commun. 2018 Oct 11;9:4223. doi: 10.1038/s41467-018-06741-w (PMC6181965; doi:10.1038/s41467-018-06741-w)
Supplement: Supplementary file 1 — Supplementary Information [file 41467_2018_6741_MOESM1_ESM.pdf]

Non-neutralizing antibodies elicited by recombinant Lassa-Rabies vaccine are critical for protection against Lassa Fever

Abreu-Mota et al.

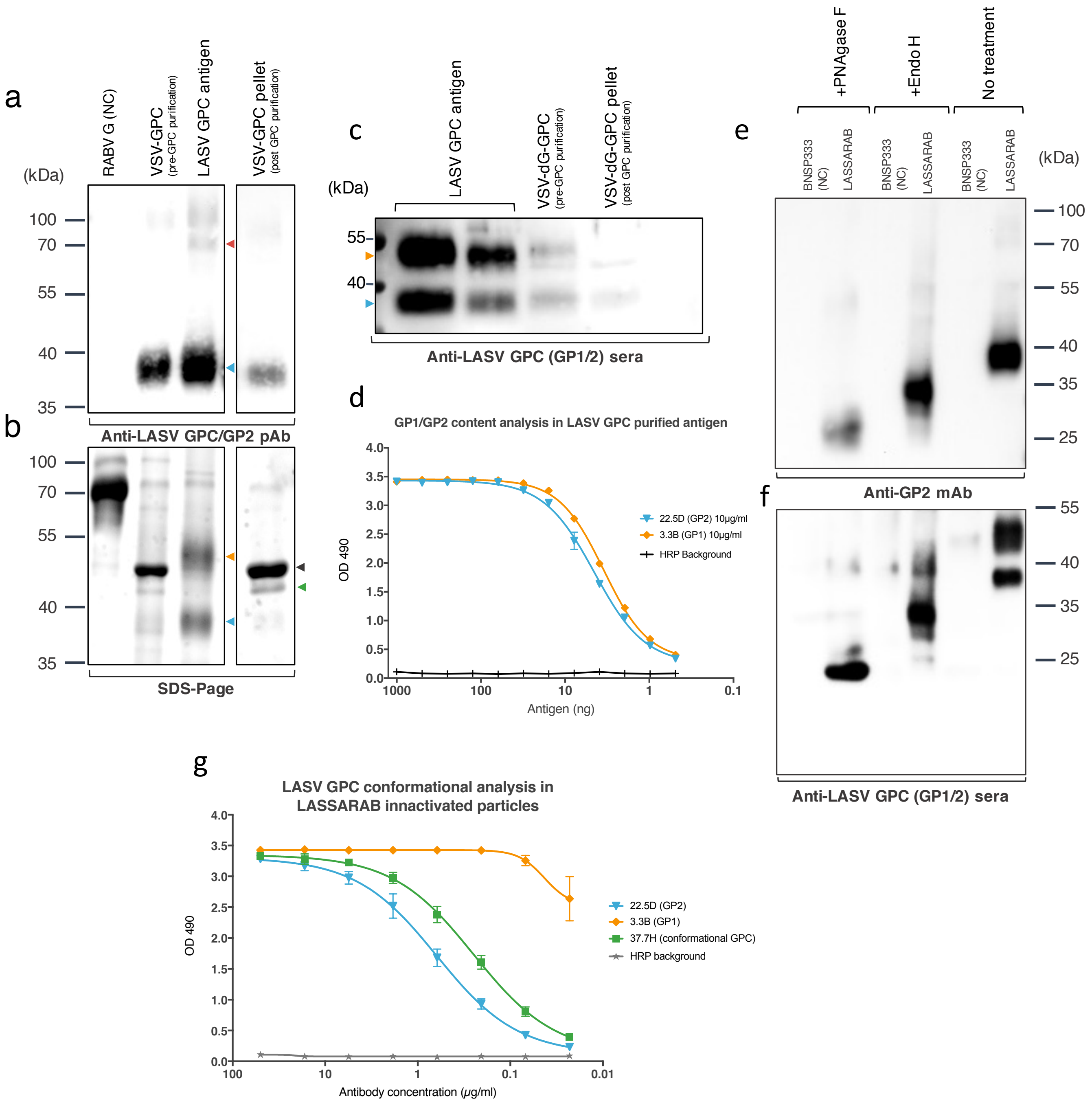
a
RABV G (NC)
VSV-GPC (pre-GPC purification)
LASV GPC antigen
VSV-GPC pellet (post GPC purification)
(kDa)
100
70
55
40
35
Anti-LASV GPC/GP2 pAb
b
SDS-Page
c
LASV GPC antigen
VSV-dG-GPC (pre-GPC purification)
VSV-dG-GPC pellet (post GPC purification)
(kDa)
55
40
Anti-LASV GPC (GP1/2) sera
d
GP1/GP2 content analysis in LASV GPC purified antigen
OD 490
Antigen (ng)
22.5D (GP2) 10µg/ml
3.3B (GP1) 10µg/ml
HRP Background
e
+PNAgase F
+Endo H
No treatment
BNSP333 (NC)
LASSARAB
(kDa)
100
70
55
40
35
25
Anti-GP2 mAb
f
55
40
35
25
Anti-LASV GPC (GP1/2) sera
g
LASV GPC conformational analysis in LASSARAB innactivated particles
OD 490
Antibody concentration (µg/ml)
22.5D (GP2)
3.3B (GP1)
37.7H (conformational GPC)
HRP background

Supplementary Figure 1. Qualitative and quantitative analysis of LASV GPC content in both ELISA GPC antigen and LASSARAB inactivated virions. (a-d) Analysis of ELISA LASV GPC antigen. rVSV-GPC purified particles (before and after LASV GPC enrichment – lane 2 and 4 respectively) and LASV GPC antigen (lane 3) were analyzed by both western blot (a) and SDS-PAGE gel stained with SYPRO ruby (b). Westen blot on (c) was probed with anti-LASV sera of survivor guinea pigs to detect both LASV GP1 and GP2. 2 µg of protein (measured by BCA) were loaded into each lane. Lane RABV G (a&b), purified RABV G antigen was loaded as a control. In lane VSV-GPC (a-c), 2 μg of sucrose cushion purified rVSV-GPC virions were loaded (pre-GPC purification). On (a-c), LASV GPC antigen lane represents 1 of the different supernatant fractions of GPC purified antigen (lipid fraction of rVSV-GPC). In (a-c) lane VSV-GPC pellet the non-lipid fraction of rVSV-GPC was loaded to confirm LASV GPC depletion. Both LASV GP1 (45 kDa-orange triangle) and LASV GP2 (38 kDa-blue triangle) can be both observed on LASV GPC antigen lane on (b) and in both LASV GPC antigen and VSV-GPC lanes on (c). In addition to LASV GP2 in (a) a 75 kDa band corresponding to LASV GPC (red triangle) is detected. An enhancement of signal for LASV GP1 and GP2 is observed from VSV-GPC lane to LASV GPC antigen lane accompanied by the disappearance (b) of VSV N and P (black and green triangles respectively) confirming LASV GPC purification. (d) LASV GP1 and GP2 presence in the antigen was further confirmed by ELISA with antigen coated at different concentrations and probed with GP2 specific 22.5D and GP1 specific 3.3B[44] .
In panels (e-g) LASSARAB incorporation of LASV GPC in inactivated particles was evaluated through western blot (e&f) and ELISA (g). Glycosylation pattern was also characterized through mobility shift assay by treating LASSARAB virions with both Endo H and PNAgase F (respective lanes). Both GP2 and GP1 have their respective reported sizes (38 kDa and 47-42 kDa respectively) in non treated conditions (e&f – no treatment lane). Upon Endo H treatment GP2 migrates to around 32 kDa (e) and GP1 shifts to a band spanning from 45 kDa to 35 kDa (f), both consistent with previous reports thereby confirming a similar glycosylation pattern as previously reported. PNAgase F treatment further shifts both GP1 and GP2 to a lower molecular size between 25 to 20 kDa. Of note, LASSARAB treatment with Endo H and PNAgase F resulted in extensive GPC aggregation with a molecular size higher than 180 kDa (shown in uncropped western blot in SD) thus diminishing the signal in western blot analysis. In (g) inactivated LASSARAB virions were coated in ELISA plates and probed for LASV GP1 (3.3B), GP2 (22.5D) and GPC (37.7H – conformational quaternary GPC-B antibody) to further confirm LASSARAB‘s LASV GPC conformational integrity post BPL inactivation. Error bars are representative of the standard error mean (SEM) of 3 replicates.

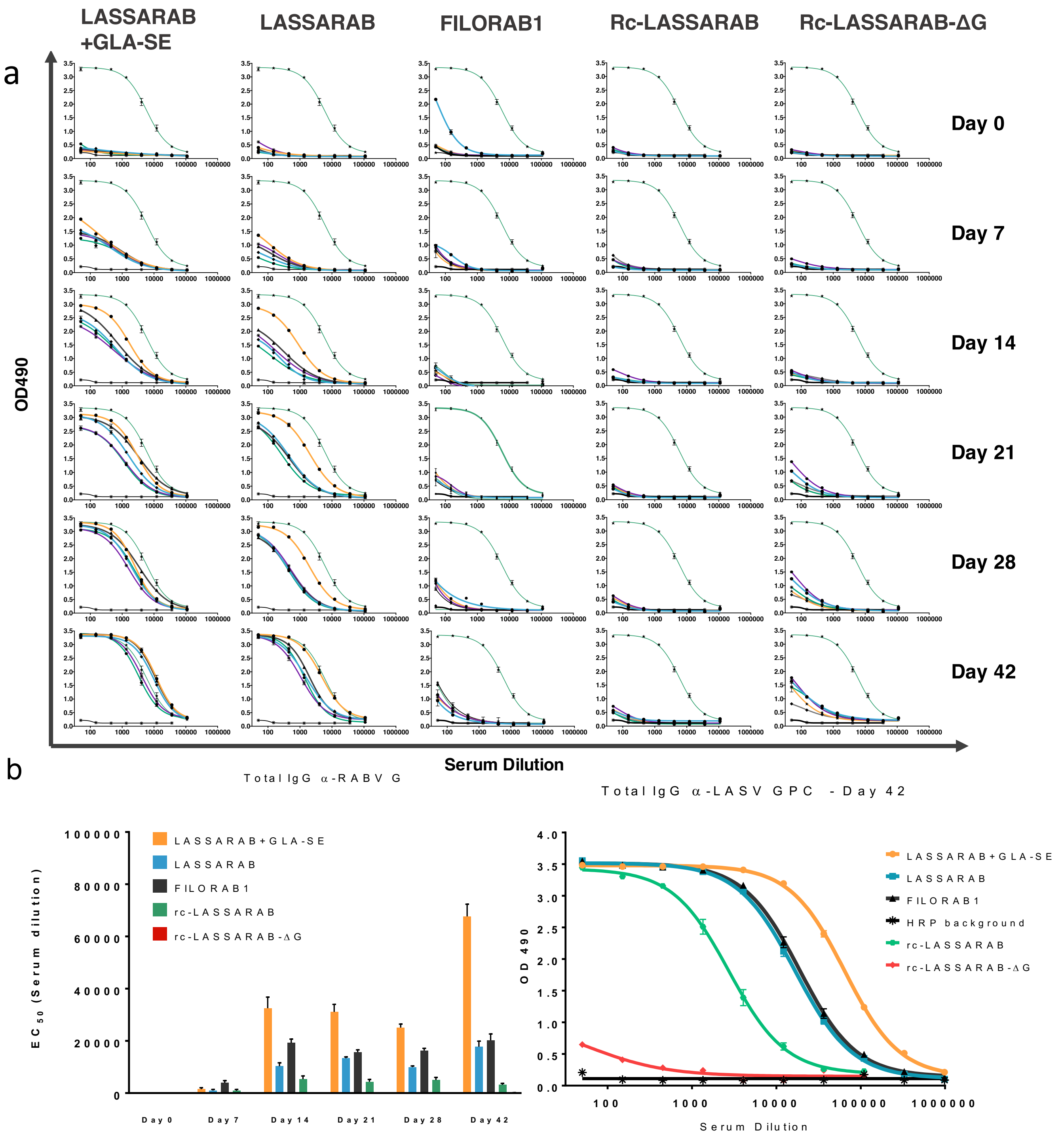

Supplementary Figure 2. IgG analysis of LASV GPC and RABV G specific IgG of mice immunized with either Replication-competent LASSARAB or LASSARAB inactivated virions (see figure 4 for experiment outline). (a) Analysis overtime of LASV GPC specific IgG at day 0, 7, 14, 21, 28 and 42. Inactivated LASSARAB+GLA-SE and LASSARAB seroconverted to LASV GPC by day 14. Replication-competent LASSARAB-ΔG had a late seroconversion at day 21. FILORAB1 immunized mice were used as a negative control. (b) At the left the average of EC_{50} values for RABV G specific IgG titers is plotted over time. All groups, except the replication-competent LASSARAB-ΔG, seroconverted to RABV G by Day 7 and reached maximum titers by Day 14 that were maintained until day 28. On day 42 titers increased after a day 28 boost for inactivated vaccines (LASSARAB+GLA-SE, LASSARAB and FILORAB1 groups). On the right ELISA curves derived from serum dilution are plotted for the indicated groups. Each symbol is the average OD490 value of individual mouse sera of each respective group. Error bars are representative of the standard error mean (SEM) of 5 mice.

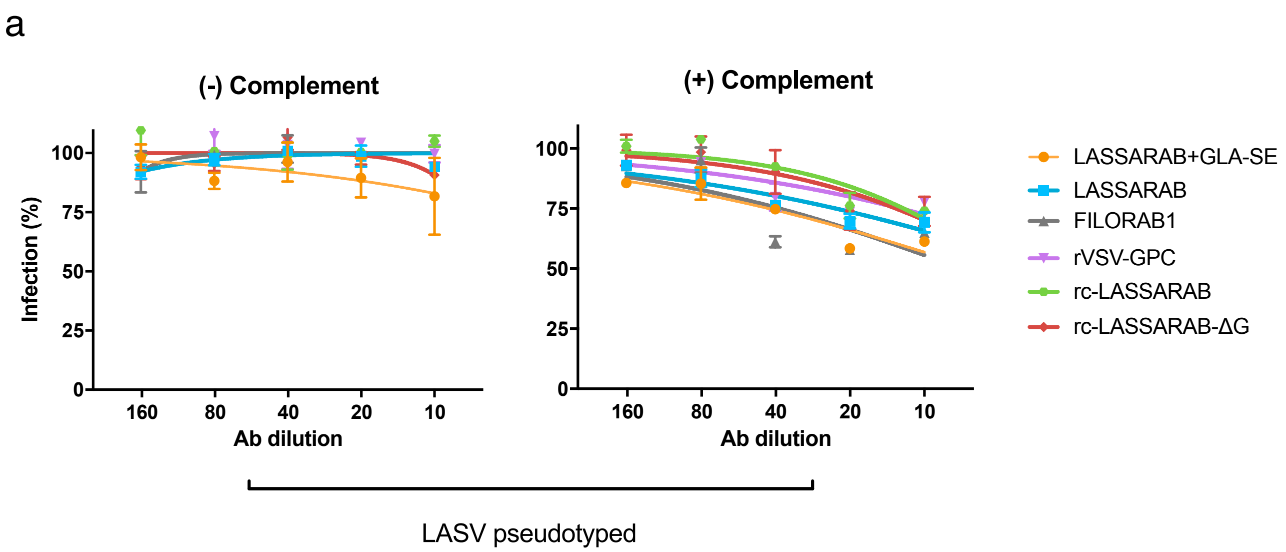

Supplementary Figure 3. Effect of addition of 10% complement on LASV neutralization in the pseudotyped LASV GPC ppVSV-NanoLuc assay. (a) Complement addition increased neutralization at lower antibody dilutions in all groups including FILORAB1 (negative control) group. Since the background neutralization was higher in the samples in which complement, was added complement addition was not further pursued in this pseudotyped virus neutralization assay. Error bars are representative of the standard error mean (SEM) of 5 mice.

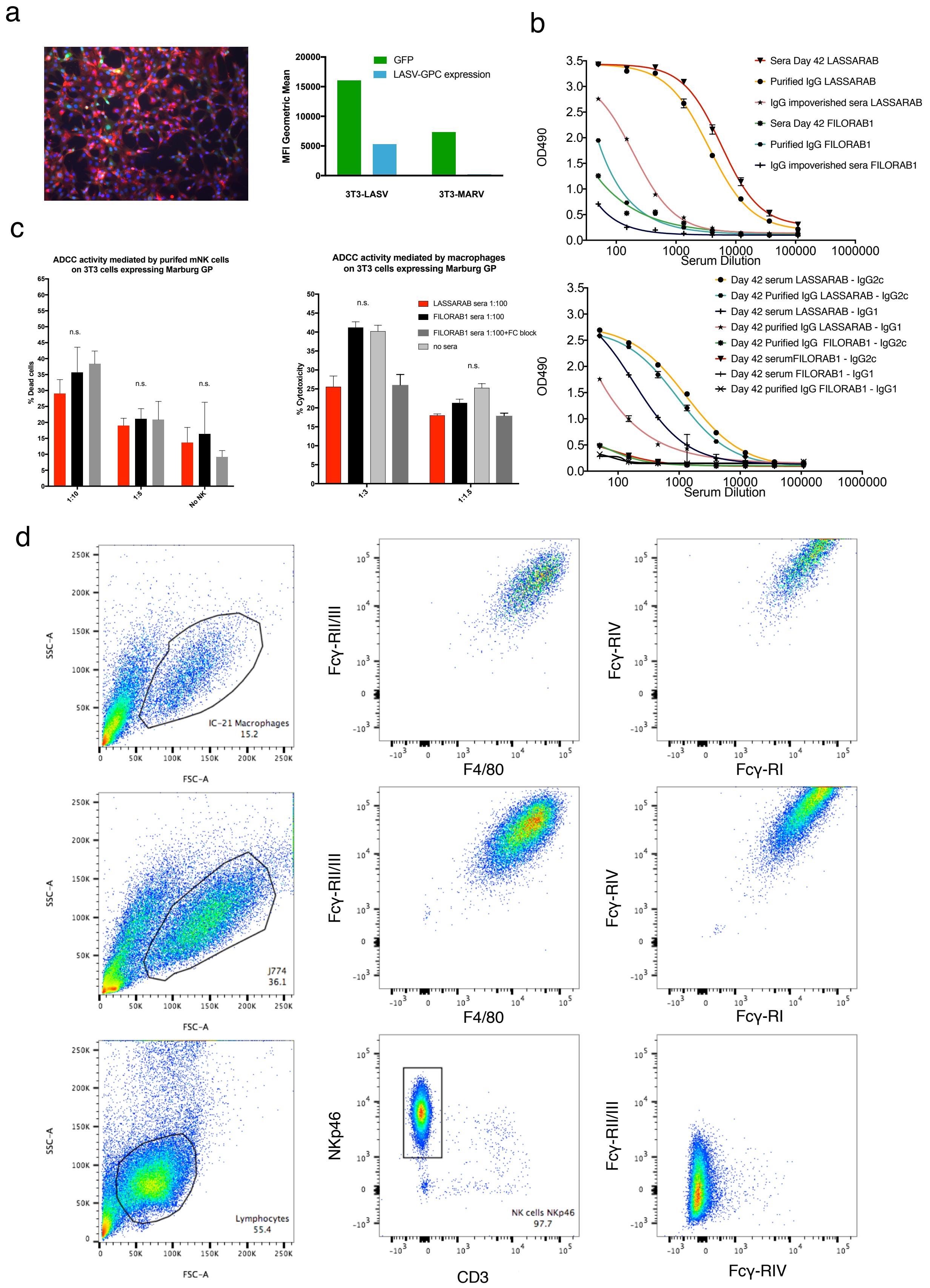
a
b
c
d
MFI Geometric Mean
GFP
LASV-GPC expression
3T3-LASV
3T3-MARV
OD490
Serum Dilution
Sera Day 42 LASSARAB
Purified IgG LASSARAB
IgG impoverished sera LASSARAB
Sera Day 42 FILORAB1
Purified IgG FILORAB1
IgG impoverished sera FILORAB1
Day 42 serum LASSARAB - IgG2c
Day 42 Purified IgG LASSARAB - IgG2c
Day 42 serum LASSARAB - IgG1
Day 42 purified IgG LASSARAB - IgG1
Day 42 Purified IgG FILORAB1 - IgG2c
Day 42 serumFILORAB1 - IgG2c
Day 42 serum FILORAB1 - IgG1
Day 42 purified IgG FILORAB1 - IgG1
ADCC activity mediated by purifed mNK cells on 3T3 cells expressing Marburg GP
% Dead cells
n.s.
1:10
1:5
No NK
ADCC activity mediated by macrophages on 3T3 cells expressing Marburg GP
% Cytotoxicity
LASSARAB sera 1:100
FILORAB1 sera 1:100
FILORAB1 sera 1:100+FC block
no sera
1:3
1:1.5
SSC-A
FSC-A
IC-21 Macrophages
15.2
J774
36.1
Lymphocytes
55.4
Fcγ-RII/III
F4/80
Fcγ-RIV
Fcγ-RI
NKp46
CD3
NK cells NKp46
97.7

e

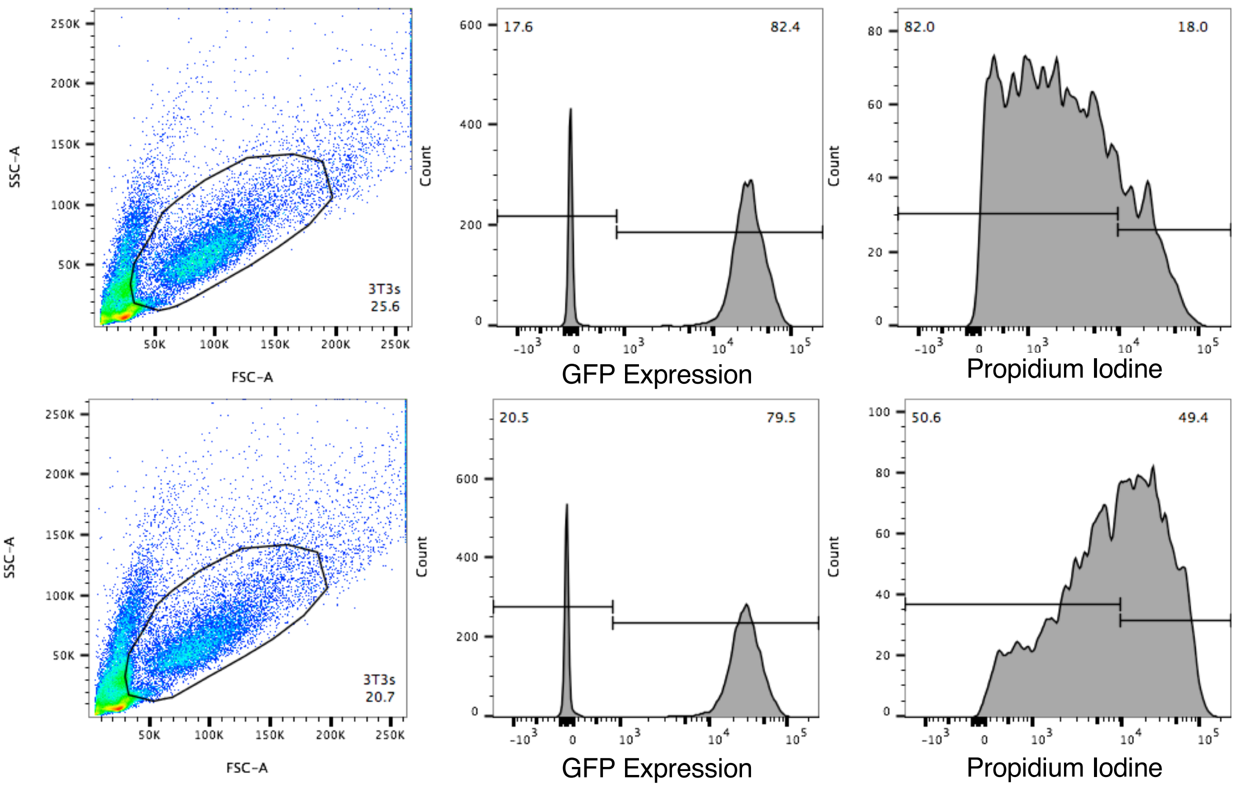
SSC-A
FSC-A
3T3s
25.6
20.7
Count
17.6
82.4
20.5
79.5
GFP Expression
82.0
18.0
50.6
49.4
Propidium Iodine

f

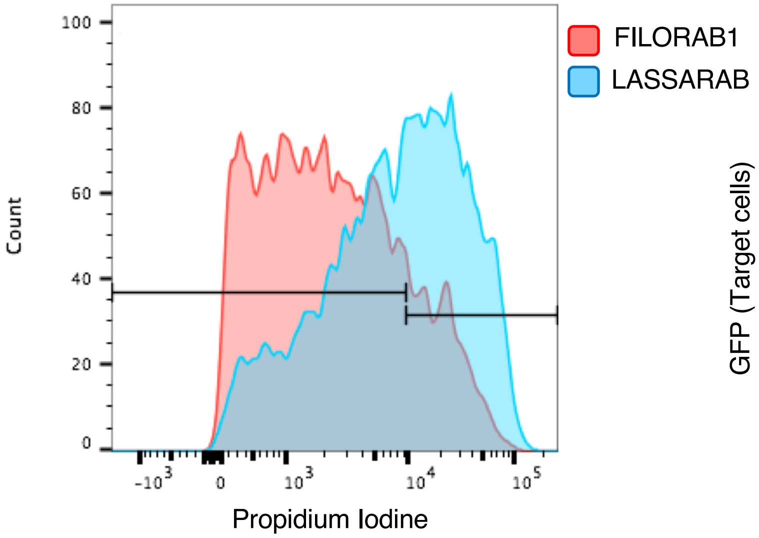
FILORAB1
LASSARAB
Count
Propidium Iodine

g

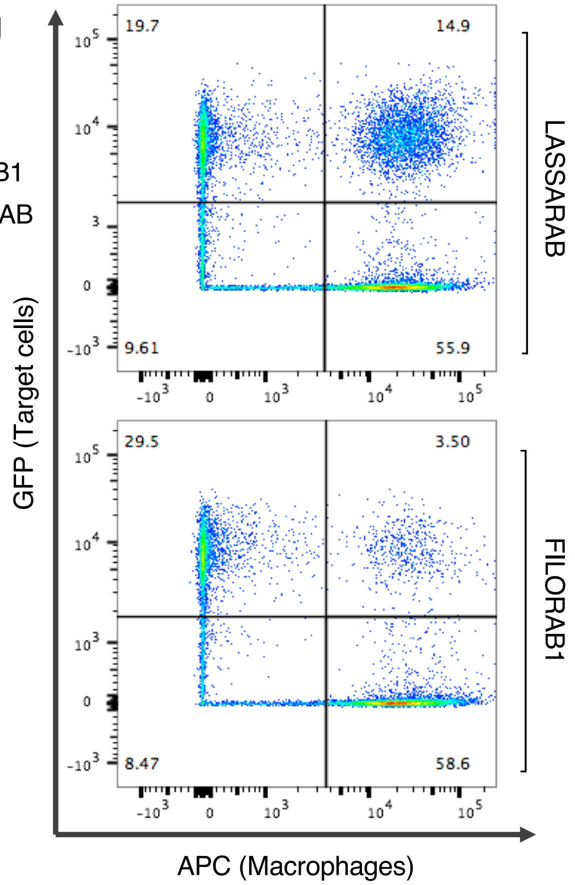
19.7
14.9
9.61
55.9
LASSARAB
29.5
3.50
8.47
58.6
FILORAB1
GFP (Target cells)
APC (Macrophages)

Supplementary Figure 4. ADCC and ADCP assay development (Figure 7). (a) LASV GPC expression in 3T3-LASV target cells was confirmed by IF (left) and Flow cytometry (right). The geometric mean of medium fluorescence intensity (MFI) was plotted in the Y axis. 3T3- MARV were used as a negative control for LASV GPC expression. (b) Day 42 total sera, IgG purified (in PBS buffer) and IgG impoverished sera from B57BL/6 mice immunized with 10 μg of inactivated LASSARAB+GLA-SE and FILORAB1+GLA-SE on day 0 and day 28 was assayed for anti-LASV GPC IgG titers. On the left graph ELISA curves are shown for total GPC specific IgG titers. On the left GPC specific IgG2c and IgG1 titers are shown. (c) ADCC and ADCP activity mediated by mNK cells and macrophages respectively against 3T3 cells expressing Marburg virus glycoprotein. No significant difference was found. (d) Characterization of macrophage and murine NK effector cells used for ADCC and ADCP assays. Top 2 rows of flow plots show gating strategy for the characterization of IC-21 and J774A.1 macrophage effector cells. Both macrophage cell lines have F4/80^{+} staining and high expression of all Fcγ-R as expected for macrophages. Bottom row shows gating strategy for murine NK cell characterization. Murine NK cells were purified from C57BL/6 mouse splenocytes using a murine NK cell isolation kit (Miltenyi Biotec). After purification murine NK cells (NKp46^{+}/CD3^{-} population) comprise 97.7% of effector cells used in the assays thus excluding potential ADCC by other effector cells (Figure 7a&b). (e) ADCC percentage gating strategy on 3T3-LASV target cells (Figure 7a, b and d). Percentage of cytotoxicity was calculated from the percentage of GFP^{+} (3T3-LASV) cells and Propidium iodine^{+} (PI). Top row is a representative flow plot of FILORAB1 sera incubated condition (control). Bottom row is a representative flow plot of LASSARAB sera incubated condition (control). (f) Overlapping PI histograms of 3T3-LASV cells incubated with either LASSARAB sera (blue) or FILORAB1 incubated sera (red) showing cytotoxicity differential. (g) ADCP flow cytometry based analysis (Figure 7c). After dead cell exclusion by viability dye, top right quadrant represents the percentage of ADCP mediated by macrophages by analyzing the percentage of GFP^{+} (target cells) and APC^{+} (Macrophages) and was plotted in (Figure 7c). In (g), top graph is a representative plot of 3T3-LASV cells incubated with LASSARAB sera, and bottom graph is a representative plot of 3T3-LASV cells incubated with FILORAB1 sera. Error bars are representative of the standard error mean (SEM) of at least 3 independent replicate experiments.

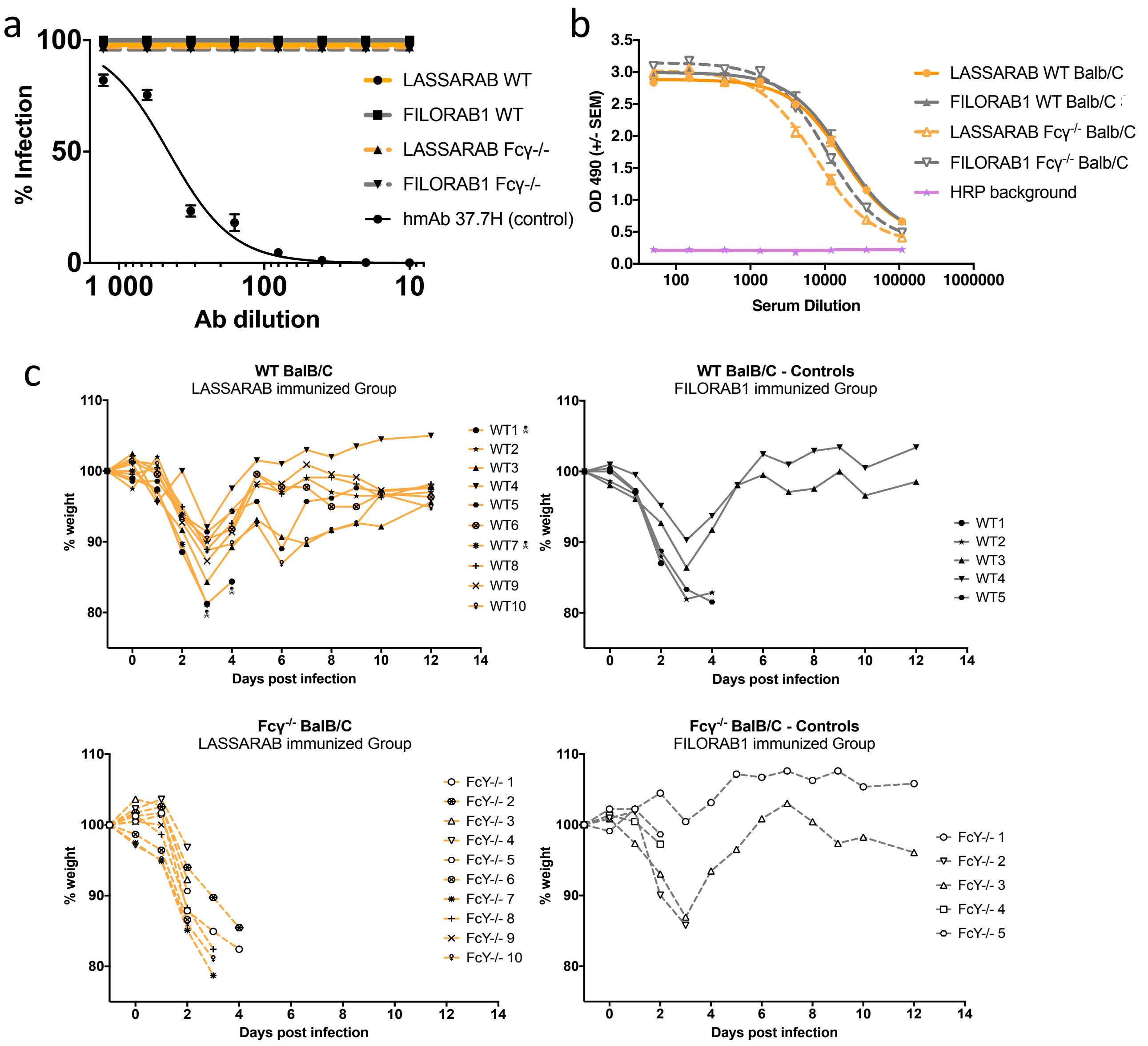

Supplementary Figure 5. *In vivo* importance of Fcγ-R functions for protection against surrogate LASV exposure (Main Figure 8). BALB/c or BALB/c Fcγ$^{-/-}$ mice were immunized with 10 μg of inactivated LASSARAB+GLA-SE and FILORAB1+GLA-SE on day 0 and day 28. (a&b) Pre-exposure (Day 35 post-immunization) analysis of neutralizing antibodies (NAbs) against LASV pseudotypes and anti-RABV G IgG titers. (a) no LASV NAbs were detected in any group prior to challenge thus excluding prior NAb protection. (b) The degree of response to RABV G is similar between all mice in all groups indicating that the lack of Fcγ does not compromise humoral response to vaccination as seen for LASV GPC antigen in the LASSARAB groups. (c) Mouse weight was recorded post surrogate LASV exposure. WT LASSARAB mice mostly resisted infection, with 2 mice dying without showing severe signs of disease. Fcγ$^{-/-}$ mice immunized with LASSARAB all succumbed to infection, with some showing severe clinical signs before weight endpoint criteria was reached. Control mice from both WT and Fcγ-/- groups all showed mild to severe signs of infection except for the FcY-/- 1 mouse (open circle dashed grey line), and 40% managed to recover from disease. All error bars are the standard error mean (SEM) of a total 20 mice for LASSARAB groups and 10 mice for FILORAB1 groups.

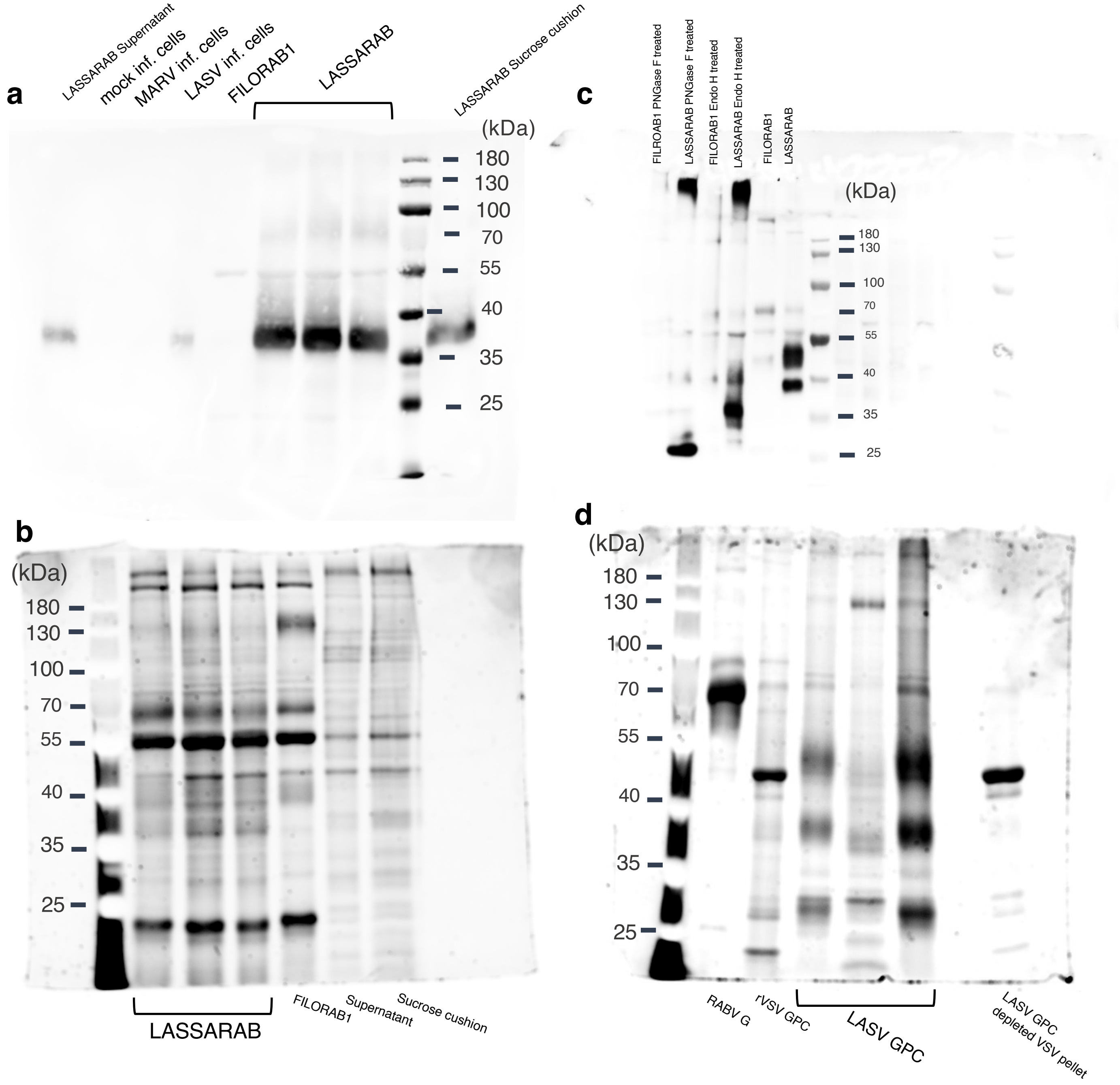

Supplementary Figure 6. Uncropped gels used to prepare Figure 2 and Supplementary Figure 1. a) Uncropped figure 2, panel e. b) Uncropped figure 2, panel d. c) Uncropped figure 2, panel f, and Supplementary Figure 1, panel f. d) Uncropped figure used in Supplementary Figure 1, panel b.

Construct	Forward Primer	Sequence 5' to 3'	Reverse Primer	Sequence 5' to 3'
LASSARAB	RP951	GGAGGTCGACTAAAGAGATCTCACATAC	RP952	TTCTTCAGCCATCTCAAGATCGGCCAGAC
rVSV-GPC	RLP3	CGATCTGTTTACGCGTGCCGCCACCATGGGACAG	RLP4	GAAGAATCTGGCTAGCTCAGCGTTTCCACTTGACT
rVSV-NL-EGFP	VP11	AGATATCACGCTCGAATGGTGAGCAAGGGCGAGG	VP12	GAAGAATCTGGCTAGTTACTTGTACAGCTCGTCCATGCC
rVSV-NL-EGFP	VP9	CGATCTGTTTACGCGATGGTCTTCACACTCGAAGATTTCG	VP10	GGATTTGAGGCGGCCTTACGCCAGAATGCGTTCGC
MSCV-GPC-GFP	MP3	CCGGAATTAGATCTCTCGAGATGGGACAGATCGTGA	MP4	AGTGGAAACGCTGAGCTAGCGAATTCCGCCCCCCCC
rVSV-GPC	VP5	GCGTGGGTCCTGGATTCTAT	VP6	ATCGAGGGAATCGGAAGAGAAT

Supplementary Table 1. Primers used for constructing and sequencing the plasmid vectors used
